# Kelvin probe force microscopy work function characterization of transition metal oxide crystals under ongoing reduction and oxidation

**DOI:** 10.3762/bjnano.10.155

**Published:** 2019-08-02

**Authors:** Dominik Wrana, Karol Cieślik, Wojciech Belza, Christian Rodenbücher, Krzysztof Szot, Franciszek Krok

**Affiliations:** 1 Marian Smoluchowski Institute of Physics, Jagiellonian University, Lojasiewicza 11, 30-348 Krakow, Poland; 2 Institute of Energy and Climate Research (IEK-3), Forschungszentrum Jülich GmbH, 52425 Jülich, Germany; 3 Peter Grünberg Institute (PGI-7), Forschungszentrum Jülich GmbH, 52425 Jülich, Germany; 4 August Chelkowski Institute of Physics, University of Silesia, 40–007 Katowice, Poland

**Keywords:** Kelvin probe force microscopy (KPFM), reduction and oxidation, SrTiO_3_, TiO nanowires, TiO/SrTiO_3_ heterostructure, transition metal oxides, work function

## Abstract

Controlling the work function of transition metal oxides is of key importance with regard to future energy production and storage. As the majority of applications involve the use of heterostructures, the most suitable characterization technique is Kelvin probe force microscopy (KPFM), which provides excellent energetic and lateral resolution. In this paper, we demonstrate precise characterization of the work function using the example of artificially formed crystalline titanium monoxide (TiO) nanowires on strontium titanate (SrTiO_3_) surfaces, providing a sharp atomic interface. The measured value of 3.31(21) eV is the first experimental work function evidence for a cubic TiO phase, where significant variations among the different crystallographic facets were also observed. Despite the remarkable height of the TiO nanowires, KPFM was implemented to achieve a high lateral resolution of 15 nm, which is close to the topographical limit. In this study, we also show the unique possibility of obtaining work function and conductivity maps on the same area by combining noncontact and contact modes of atomic force microscopy (AFM). As most of the real applications require ambient operating conditions, we have additionally checked the impact of air venting on the work function of the TiO/SrTiO_3_(100) heterostructure, proving that surface reoxidation occurs and results in a work function increase of 0.9 eV and 0.6 eV for SrTiO_3_ and TiO, respectively. Additionally, the influence of adsorbed surface species was estimated to contribute 0.4 eV and 0.2 eV to the work function of both structures. The presented method employing KPFM and local conductivity AFM for the characterization of the work function of transition metal oxides may help in understanding the impact of reduction and oxidation on electronic properties, which is of high importance in the development of effective sensing and catalytic devices.

## Introduction

Transition metal oxides are viewed today as some of the most promising materials in various fields, ranging from (photo)catalysis [1], hydrogen production [2], resistive switching [3] and organic electronics [4–5] to so-called thermoelectric power generators [6]. The performance of all of the abovementioned applications is extremely sensitive to the work function (WF) of the active oxide layer. As a vast majority of applications are nowadays based on oxide heterostructures, not only is macroscopic information of the work function needed (which may be provided by averaging techniques such as ultraviolet photoelectron spectroscopy (UPS)) but also spatial resolution on the nanoscale. Driven by its remarkable lateral and energetic resolution, Kelvin probe force microscopy (KPFM, also known as scanning Kelvin probe microscopy, SKPM) is the tool of choice for the precise measurement of the WF across oxide heterostructures, which is a technique that has not been fully exploited to date. In recent years, KPFM has proved to be superior for many cases in both fundamental research and applications, such as the identification of adsorption geometries of molecules on oxide surfaces [7], probing energetics of electron transfer within single molecules [8] and operation of prototypical electronic devices, such as perovskite solar cells [9] or Ti/TiO*_x_*/Ti memristive devices [10]. Of the two KPFM operation modes, frequency modulation (FM) has proven to be more suitable for the investigation of oxide nanostructures (due to the higher lateral resolution) as compared to amplitude modulation (AM) [11]. Therefore, in our study, we present the advantages and limitations of the FM-KPFM technique using the example of a newly discovered TiO/SrTiO_3_(100) (metal/insulator) heterostructure, which has potentially high technological relevance [12].

Now it would be justified to introduce both TiO and SrTiO_3_ oxides, highlighting the differences and similarities between those two structures. Based on the electronic conduction, most transition metal oxides could be classified as insulators or semiconductors. However, due to the plethora of available valence states in which a cation can be, many transition metal oxides may also exhibit metallic conductivity. Here, a huge advantage over other materials is the possibility of oxides that self-dope via the introduction of oxygen vacancies [13], which is also a reason why there are not many reliable experimental studies on the work function of transition metal oxides (with one notable exception [14]). Strontium titanate, SrTiO_3_, is a perfect example of a semiconductor with a wide bandgap of 3.2 eV and also a model perovskite oxide. Ti^4+^ cations provide no electrons for the d-band, which can participate in conductivity. Strontium titanate finds many applications as a dielectric ceramic material [15] but also in various heterostructures, with exotic electronic states, e.g., a two-dimensional electron gas (2DEG) on the interface with LaAlO_3_ [16–17]. On the other side of the transition metal oxides spectrum lies titanium monoxide, TiO, in which the titanium atoms adopt the valence state 2+, contributing to the formation of d^2^ electrons. Hence, TiO is a d-band conductor with a room temperature conductivity of 3500 Ω^−1^ cm^−1^, which slowly decreases with temperature [18]. There is a growing interest in such metallic oxides, not only as oxide electrodes but also in medicine, since reduced TiO nanostructures exhibit a strong absorbance of light over a broad spectrum, which is of potential use in novel tumor therapy [19]. In addition, in the previous year, a profound interest was focused on the superconductivity of various TiO structures [20–21]. According to those, rock-salt TiO is a type-II superconductor with a superconductivity transition temperature (*T*_c_) of 5.5 K, which is higher than previously reported results [22]. As a result of its electronic structure, titanium monoxide nanoparticles find further application in heterogeneous catalysis, e.g., for the hydrogenation of styrene [1].

Here we show the properties of a bulk-like crystalline rock-salt TiO phase, unlike previous studies on the crystallography and electronic structure of TiO, which were based on defective thin films formed on various surfaces, e.g., TiC(100) [23] or TiO_2_(110) [24]. γ-TiO is the high-temperature phase with a NaCl-type (rock-salt-type, B1) cubic structure (*Fm*−3*m*, *a* = 4.184 Å), displaying a wide range of nonstoichiometric values [25]. Our study contains first measurements of electrical conductivity and the work function of crystalline TiO and its response to the ambient air reoxidation. This is all compared to the work function of another relevant oxide, SrTiO_3_(100), showing similarities and differences.

The present manuscript is organized as follows: after the introduction of our TiO/SrTiO_3_(100) system, a combined conductivity and work function study from the same surface area is presented, showing the possibility of obtaining full information on the electronic properties when the KPFM technique is accompanied by local conductivity atomic force microscopy (LC-AFM). This is followed by a discussion of the significant variations of the WF within cubic TiO nanowires, the estimation of the KPFM resolution and the differences between TiO_2_ and SrO terminations of SrTiO_3_(100). The last part of the study is dedicated to the discussion of the work function response of both TiO and SrTiO_3_ surfaces upon oxidation via ambient air exposure, in order to provide insight into the effect of oxygen, water, and carbon dioxide interaction and therefore mimic the operation conditions in real life applications.

## Results and Discussion

**Figure 1:**
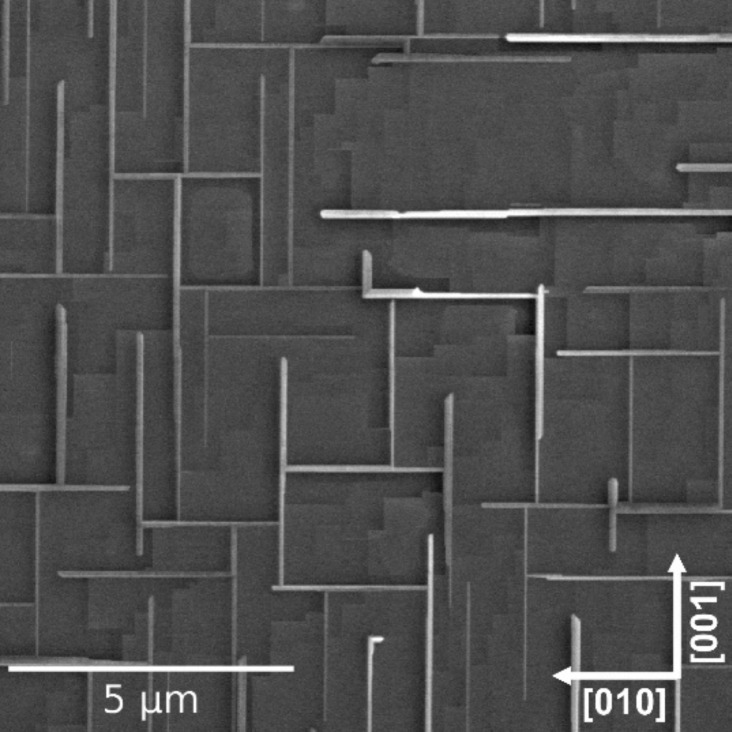
SEM images of an oriented network of titanium monoxide (TiO) nanowires on SrTiO_3_(100). In between the terrace structure of SrTiO_3_(100) can be seen.

The thermal reduction of a SrTiO_3_(100) crystal under reduced oxygen partial pressure (UHV conditions + an oxygen getter), assured by the extremely low oxygen partial pressure (ELOP) process, results in the formation of a network of ordered TiO nanowires on the (100) surface as shown in Figure 1. Heavily reducing conditions enable not only the removal of oxygen from the crystal but additionally trigger an incongruent sublimation of strontium, resulting in the titanium enrichment of the surface [26]. This general process has also been observed for the broader class of transition metal oxides, such as CaTiO_3_ or BaTiO_3_. The titanium monoxide surface layer crystallizes in the form of nanowires oriented along the main crystallographic directions of SrTiO_3_(100), with a length of up to 10 µm, a width of a few hundred nanometers and a height of tens of nanometers – see Figure 1. Their size could be easily tuned either by the temperature or time of reduction [12]. The perfect crystallographic order of TiO nanostructures is confirmed by the transmission electron microscopy (TEM) measurements in the high-angle annular dark-field imaging scanning transmission electron microscope (HAADF-STEM) mode as we have recently shown in [12]. A closer look at TiO reveals the γ-TiO structure in a Fm-3m space group and 2.1 Å unit cell, which stands in perfect agreement with subject literature values [27]. The high level of crystallinity of these nanowires is the result of the formation mechanism, which comprises incongruent strontium effusion from SrTiO_3_ and then a TiO structure growth via crystallographic shearing and diffusion (more details can be found in [12]). The abrupt TiO/SrTiO_3_ interface and the Ti^2+^ to Ti^4+^ transition thereof make such a transition metal oxide heterostructure a promising candidate for various electronic properties and charge transfer investigations.

**Figure 2:**
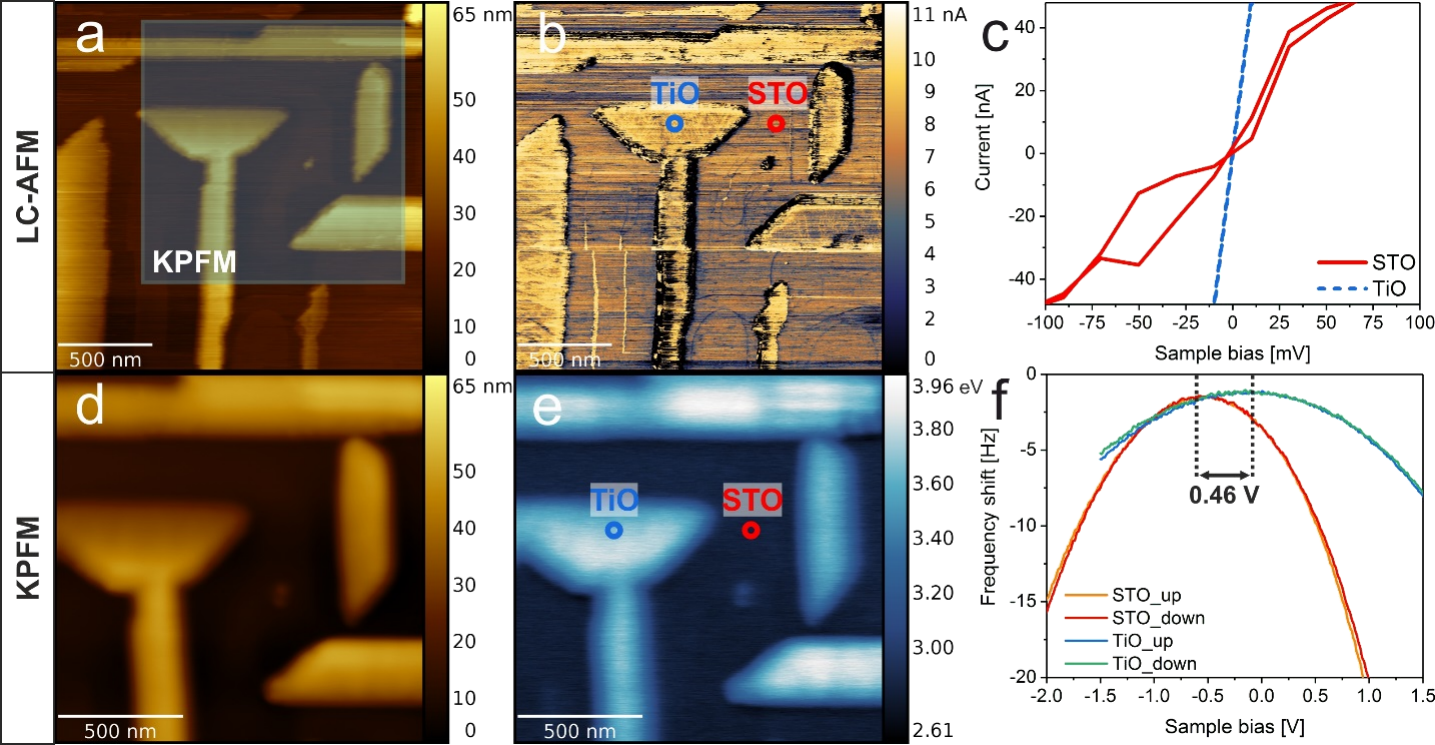
Electrical properties of TiO/SrTiO_3_(100) heterostructures. a), b) LC-AFM topography and current (PtIr-coated PPP-ContPt tip, +1 mV sample bias), c) *I*–*V* characteristic (up and down sweeps) of marked areas on a TiO nanowire and SrTiO_3_ (STO) surface, d,e) KPFM topography and work function measurement of the very same area, and f) Kelvin parabola at the same TiO and STO structures.

Despite the similarities (both structures have a cubic crystallographic phase), there are profound differences in the electronic structure between the band insulator SrTiO_3_ and metallic TiO. Here there are two 3d electrons per one Ti^2+^ divalent titanium ion, partially filling the metallic d-band in the energy diagram. From the orbital perspective, high conductivity is a consequence of the d-orbital overlap from the neighboring Ti sites. In the case of cubic TiO, the Ti–Ti distance is slightly above 2 Å, which is enough to have a significant overlap given the d-orbital extension. On the other hand, in the ideal SrTiO_3_ perovskite there are no d-electrons on Ti sites. Thus a TiO network on SrTiO_3_ constitutes a metallic nanowire array embedded in an insulator matrix, and to properly disentangle the electronic properties of both structures a technique with nanoscale resolution is needed. Indeed with the use of the LC-AFM technique, the conductivity of the developed nanostructures at the nanoscale can be characterized, providing the possibility of obtaining current maps as well as *I*–*V* characteristics at a given spot. Figure 2a and Figure 2b show the topography and current maps of the TiO nanowire network on SrTiO_3_(100). TiO has a higher conductivity than the surrounding SrTiO_3_ (STO) surface (the no current areas at nanowire edges are due to technical artefacts, such as wear of the coating of the conductive probe). To better illustrate the differences, *I*–*V* characteristics of TiO and STO were collected and are presented in Figure 2c. Given the ohmic behavior at the TiO nanowire, the conductance of the whole system (tip + contact + TiO nanowire + interface + STO bulk + bottom electrode) can be estimated to *G*_LCAFM_ = 10 µS. In contrast, the STO surface exhibits a one order of magnitude lower current of a rather semiconducting nature. It is noteworthy that the STO(100) surface had been thermally reduced up to 1150 °C in UHV beforehand, resulting in the formation of a high concentration of oxygen vacancies. Therefore, the conductivity is much higher than that of a pristine single crystal, which has been estimated via a comparable LC-AFM study to be around 10^−16^ S [28]. Here the observed changes of conductivity on the surface correlate directly with the work function differences as provided by the KPFM measurements taken at the very same area. This was possible by forcing the same conductive contact AFM tip to oscillate at higher harmonics to enter the FM-KPFM mode (more details can be found in [29]). Figure 2d,e presents the topography and work function of the same area. Differences in the WF are as high as 900 meV between TiO and SrTiO_3_, in favor of TiO. However, there is also a certain variation within the TiO and SrTiO_3_ structures, which will be discussed later. The bias sweep measurements presented in Figure 2f show reproducible Kelvin parabola with negative curvature for both structures. As up and down bias sweeps appear to follow the same curve, and there is no sign of charging or charge transfer. A comparative study of LC-AFM and KPFM of the TiO/SrTiO_3_ structure enables a clear distinction between two materials of comparable conductivity but notably different work functions. The reason behind this is that although undoped SrTiO_3_(100) is a band insulator, it could be easily self-doped with oxygen vacancies upon thermal reduction [28,30]. Reduction preferentially occurs at the surface, resulting in the reconstruction transformation from (1×1) to (√5×√5)R26.6°, and in the vicinity of extended defects in a crystal (dislocations) which act as easy conduction paths for electrons. Oxygen vacancy formation, and therefore Ti^3+^ valence, results in the appearance of new t_2g_ electron states within Ti 3d, which are below the conduction band of SrTiO_3_(100) [31]. Consequently, a decrease of the WF is expected, as it was previously reported for 900 °C thermal annealing under UHV, where the WF of SrTiO_3_(100) yielded 3.478(64) eV [30]. Hence, here a high conductivity of the reduced SrTiO_3_(100) is measured and a work function of 3.12(18) eV, which is almost 1 eV lower than previous X-ray photoemission electron microscopy (XPEEM) and UPS studies (4.13 and 4.2 eV) for untreated oxide [32–33].

**Figure 3:**
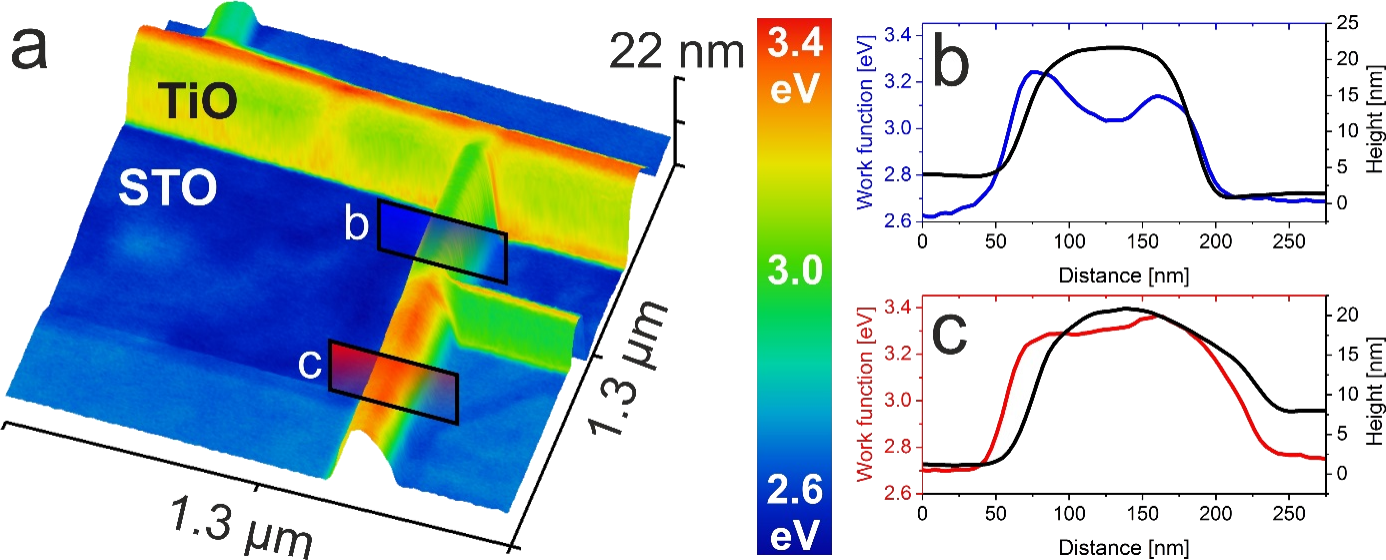
TiO facets influence the work function (WF) on the nanoscale. a) 3D view of the combined topography and the WF of TiO/SrTiO_3_. b,c) Topography and work function profiles of two areas on the same TiO nanowire.

As stated before, KPFM investigations reveal certain variations in the work function value of TiO nanostructures. To illustrate this properly a 3D topography overprinted image with color scale representing the WF is shown in Figure 3a. The WF of titanium monoxide varies to as high as 300 meV, even within one nanowire. This is not an imaging artifact but rather a morphological-related feature, which is proved by the two profiles obtained for the same nanowire and shown in Figure 3b,c. The edges of the nanowires have a different work function from the top surface. This could be a consequence of the different facets of the cubic TiO crystal being exposed. Such an effect of facets having different WFs has to date been observed for many structures – the differences could be as high as 255 meV measured in the case of 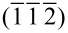 and (110) surfaces of CuGaSe_2_ [34]. For the case of transition metal oxide crystals, XPEEM studies have proved that the WF of the SrTiO_3_(111) face is higher than that of the (100) face by no less than 210 meV [32]. Smaller differences in the range of 70 meV were reported in the case of titanium dioxide (110) and (100) faces [35]. In the present case, the differences could be higher because the whole sample with TiO nanostructures was annealed up to 1150 °C and thus a possible reduction-driven non-stoichiometry occurs. From a geometrical perspective, the low index faces of cubic TiO, like (100), (110) or (210), have an equal number of protruding oxygen and titanium atoms, in contrast to the (111) face, where a whole plane is formed by either titanium or oxygen. To our knowledge, there are no subject literature studies on the stability or work function on such faces, but it is justified to assume certain differences may be present between those facets, which are also influenced by the preferential removal of oxygen during thermal reduction. As TiO nanowires have a height of tens of nanometers and tip convolution may play a role, a precise evaluation of the steep facets of a higher WF, as seen in Figure 3, is difficult. Based on the relative slope of a TiO surface it seems that the top surface typically adopts a (100) plane, though this requires further investigation.

**Figure 4:**
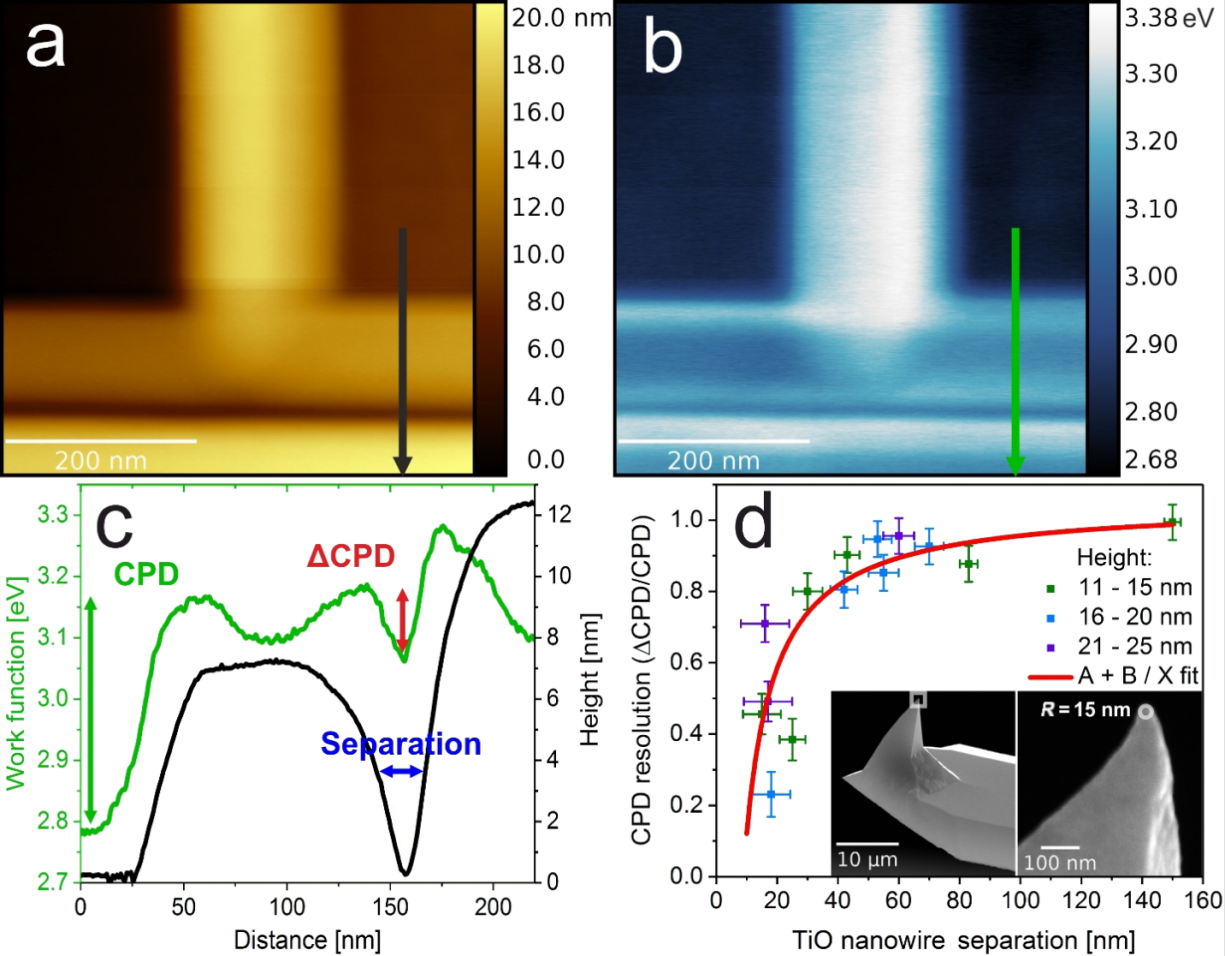
KPFM lateral resolution on high TiO/STO structures. a) Topography and b) work function of TiO nanowire array on SrTiO_3_(100). c) Height (black line) and work function (green line) profiles of two adjacent TiO nanowires, showing high KPFM contrast. d) Dependence of the CPD resolution (estimated as ΔCPD/CPD, see c) on the separation between TiO nanowires, with A + B/X asymptote fit. Insets show the SEM images of the actual PtSi cantilever used in the experiments with a tip radius of 15 nm.

An overlay of the work function on the topography map shown in Figure 3a provides additional information on the high lateral contrast of KPFM. The WF and morphology match almost completely, although height variations are of tens of nanometers. To investigate the resolution limits of KPFM imaging of oxide heterostructures with a complex topography, a set of images of parallel TiO nanowires was investigated, and the results are presented in Figure 4. As a measure of the potential resolution, we have used the ratio between the measured contact potential difference (CPD) decrease in between the TiO nanowires and the full CPD of TiO with respect to STO. For an ideal case, when the CPD reaches the value of STO in the gap between parallel nanowires, this measure adopts 1, and 0 if there is no drop in CPD. The dependence of this value on the TiO nanowire separation (plotted in Figure 4d) provides information on the resolution limit. It follows an asymptotic behavior, with the correct CPD values measured when TiO nanowires are separated by more than 40 nm. This stands in perfect agreement with the real tip radius of 15 nm, which was measured for the same cantilever in high-resolution SEM (see Figure 4d insets). Here, the use of uniformly PtIr-coated tips enables topography correlated artifacts to be avoided, unlike with some previous studies [36]. Taking into account the opening angle of about 40° and the average nanowire height of 18(8) nm, the tip diameter at the level of the top surface of the nanowire would be about 40 nm, meaning that the CPD resolution approaches the topography resolution, which is the ultimate physical limit of the FM-KPFM technique [11]. Similar results were obtained for the resolution estimation for KBr nanoislands of monoatomic thickness, where a resolution of 0.5 was obtained for 20 nm separation, as is the case in the following study [37]. The CPD resolution at the TiO/SrTiO_3_ transition is insensitive to the nanowire height, following the same asymptotic behavior (see Figure 4d), unlike the situation for C60 islands on HOPG, where a spread of 50 nm was reported, while the topography resolution was 10 nm [38]. From our results, we can set the limit of the smallest separation of oxide nanostructures at 15 nm to obtain any CPD difference. As for the CPD resolution, the estimated value would be below 5 meV, due to the high mechanical stability and good conductivity of both platinum silicide and PtIr-coated tips.

Apart from the work function difference between the TiO and STO materials, we also found that the CPD/WF mapping of the STO(100) itself exhibits a nonuniform nature. This could be associated with two different exposed surfaces, as SrTiO_3_(100) perovskite structure has two stable nonpolar terminations, SrO and TiO_2_, which both are present on the pristine surface, although TiO_2_ is more stable [39]. When annealed under reducing conditions, the TiO_2_ termination is promoted. Upon heavy reduction, there is further oxygen depletion which results in the formation of the (√5×√5)R26.6° reconstruction, which we recently proved to be a Ti-enriched layer on TiO_2_-terminated SrTiO_3_(100) [30,40]. The electronic structures of both terminations are much different – the DFT calculated work function values are 1.92 eV and 4.48 eV for SrO and TiO_2_, respectively [14], obtained for the pure surfaces without vacancies.

**Figure 5:**
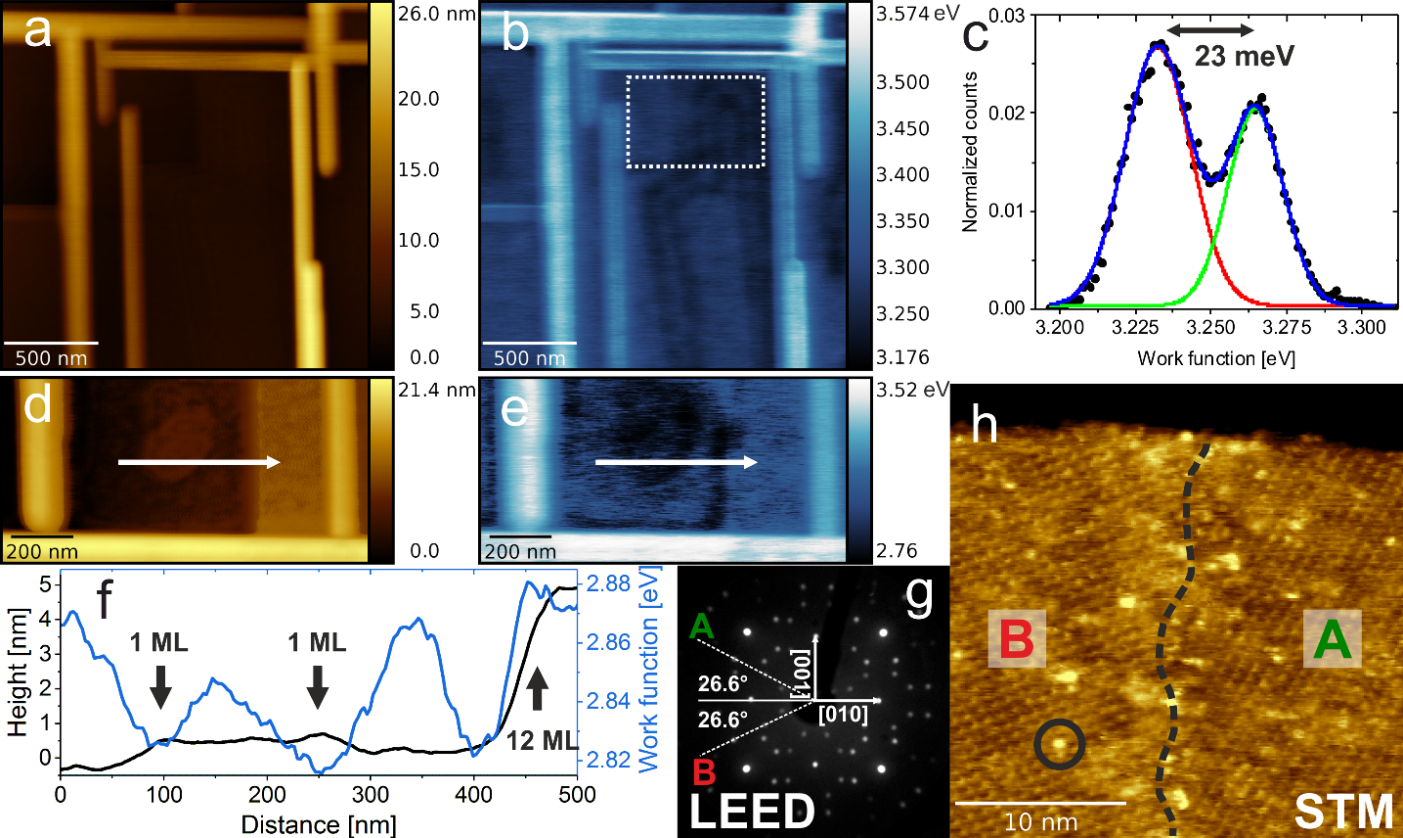
Characterization of the SrTiO_3_(100) surface by KPFM. a) Topography of a TiO/SrTiO_3_ structure (Δ*f* = −20 Hz), b) corresponding work function map from KPFM, c) work function histogram from the selected area in b), d) and e) topography and work function of a different TiO/STO network (Δ*f* = −20 Hz), showing the WF correlation with step edges. f) Topography and work function line profiles, extracted from d) and e). g) LEED pattern (100 eV beam energy) indicating the perfect (√5×√5)R26.6° reconstruction with two equiprobable domains A and B. h) STM topography showing the concurrent presence of A and B domains, with defects marked in black.

In the present case of thermally reduced SrTiO_3_(100), the dominant reconstruction is (√5*×*√5)R26.6°, which forms on the TiO_2_ termination, as proved by the scanning tunneling microscopy (STM) and low-energy electron diffraction (LEED) investigations (see Figure 5g,h). The surface is composed of two equiprobable orientations of (√5*×*√5)R26.6° domains, both rotated with respect to the (1*×*1) surface by 26.6 degrees. Those two domains are labeled A and B, both in LEED and STM images. Bright protrusions seen in the STM picture, which also decorate domain boundaries, are either oxygen vacancies/vacancy clusters or Sr adatoms, according to the model [30]. The KPFM map reveals a non-homogeneous landscape of the work function – higher values are measured on terraces, whereas areas near the edges a reduced work function is observed (see Figure 5b). This could be directly represented by the histogram shown in Figure 5c. A clear difference of ≈23 meV could be measured between the two areas. This effect is not purely topographical, since the WF differences are similar in the cases of a single step and multistep (12 atomic layers), as shown in Figure 5d–f. Three possible mechanisms are possible to cause such difference: termination-, reconstruction- or reduction-related. The first would be the termination variation within one terrace, with the higher WF values related to the TiO_2_ termination, which is dominant, and the lower to the SrO-enriched terrace edges, as previous KPFM studies showed the difference between air-annealed SrTiO_3_ terminations of ≈10 meV [41]. However, for the case of heavily reduced SrTiO_3_(100) the only observed termination is TiO_2_, given the step uniform heights of ≈4 Å. The second possible origin of the observed variation is the presence of multiple surface reconstructions, notwithstanding that the recorded LEED patterns and STM maps do not support such claims, showing clear (√5×√5)R26.6° surfaces, even near the step edges (see Figure 5h). Finally, this difference is not likely to be caused by the electronic layer at step edges since it stretches away from the edges up to 100 nm, which is too far to be attributed to local dipoles [42]. Simultaneously the oxygen removal is likely to be initiated at step edges, as it was as postulated by Martinez et al. [43] for a similar TiO_2_(110) oxide. Hence for the severe reduction of SrTiO_3_(100) a gradient of the average cation valence across one terrace may be expected, which can contribute to the observed small decrease in the WF near the step edges. In terms of the absolute work function of TiO_2_-terminated SrTiO_3_(100), the obtained value of 3.12(18) eV, averaged across a number of images, is close to this postulated by DFT (3.39 eV) for TiO_2_ termination with oxygen vacancies in the surface layer [14]. The difference is most likely caused by the higher density of oxygen vacancies, due to the heavy thermal reduction.

### Ambient air reoxidation of TiO and SrTiO_3_

As the vast majority of applications of transition metal oxides is related to the oxygen activity and redox reactions on surfaces, the next experiment was aimed to study the work function dependence upon controlled reoxidation of reduced oxides.

Transition metal oxide nanostructures find manifold applications, especially in various (photo)catalytic processes, e.g., water splitting [1,44]. For industrial uses, the samples have to be exposed to ambient conditions, therefore it is necessary to investigate the impact of air exposure on electronic surface properties. Moreover, transition metal oxides are often regarded as promising materials for sensing applications, due to the strong response to gaseous pressures, especially oxygen and water [45]. The tool of choice that provides information on the subtle changes in the work function is KPFM, which has been employed for research on the photocatalytic activity of TiO_2_-based doped and undoped nanofibers [46–47] and for the understanding of the operation of nanostructure electrochemical sensors [48].

**Figure 6:**
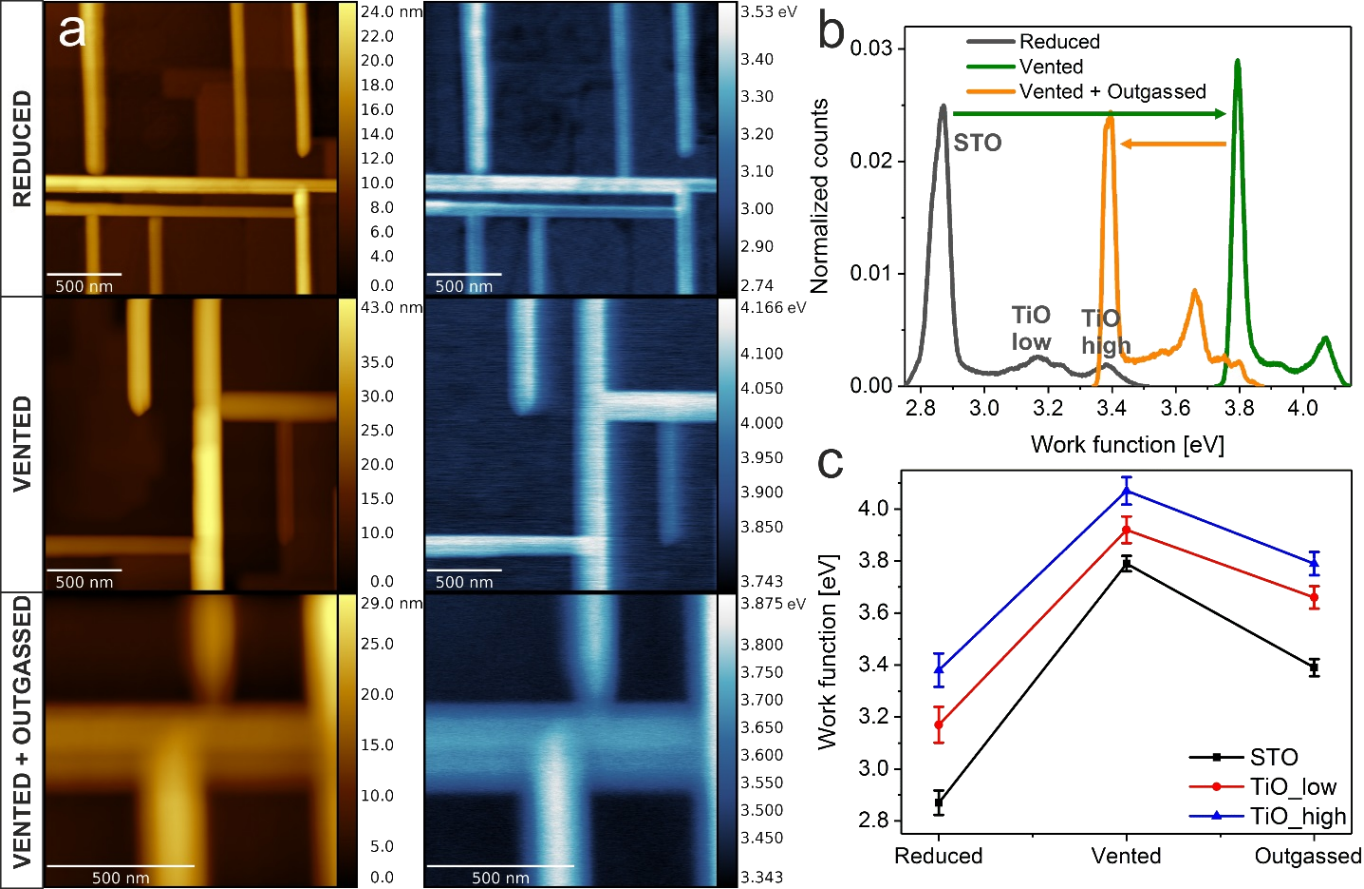
Influence of air exposure on the TiO/SrTiO_3_ work function. a) KPFM topography and work function of a TiO/SrTiO_3_ heterostructure (Δ*f* = −40 Hz) after reduction (first row), exposed to ambient air (second row) and after annealing up to 230 °C (third row). b) Work function histograms of the presented cases. c) Evolution of the work function value for the SrTiO_3_(100) surface (black), lower TiO facets (red) and higher (blue).

Thus, we have focused on the systematic approach of the influence of ambient air on the work function of TiO and SrTiO_3_(100). Figure 6a shows the topography and work function of representative TiO nanowire networks before and after ambient air exposure. Additionally, the impact of low temperature annealing (230 °C) in UHV is presented. After the venting of the vacuum chamber, the TiO nanowires still had a much higher work function than the SrTiO_3_(100) surface; however, the absolute values for both materials increased. It is widely known that water molecules present in the ambient air form a dipole layer, which can give rise to an effective surface potential [49].

To reduce the impact of weakly interacting adsorbates, in the next step, the sample underwent in situ UHV annealing, which implicated a WF decrease of both TiO and SrTiO_3_ by about 0.2–0.4 eV. To illustrate the experimental findings better, three histograms of reduced, air-oxidized and outgassed work functions are plotted in Figure 6b. It is immediately visible that the general three-peak nature of STO and lower and higher TiO values of the WF are preserved, however the offset changes, as illustrated in Figure 6c. The STO(100) work function increases by 0.9 eV due to air exposure, similar to the case of oxygen reoxidation [30]. A conclusion can be drawn that the main contribution of the work function increase is related to oxygen, which refills vacancies of the reduced (√5×√5)R26.6° surface and locally oxidizes titanium cations to regain the 4+ valence state.

Vented TiO nanowires have a work function that is higher by 0.65 eV, independent of the face, which is almost 0.5 eV higher than in the case of pure oxygen (not shown). This is a footprint of the high catalytic activity of the TiO surface, which was previously postulated in the case of TiO/TiO_2_ nanoparticles [44]. Reduced titania TiO_1.23_ proves also to be a promising candidate for electrochemical water splitting [50]. As the catalytic activity of a crystalline rock-salt TiO phase has yet to be studied, this is a first indirect observation of the high potential of such a structure.

After in situ annealing to 230 °C, the work function of TiO nanowires drops by 0.2 eV, in contrast to the 0.35 eV decrease for the SrTiO_3_(100) surface. A temperature of 230 °C was chosen to enable desorption of the most abundant adsorbates, also mimicking the typical operating temperatures of metal oxide gas sensors [51]. Numerical calculations suggest that H_2_O and CO_2_ species present in air saturate almost all free adsorption sites on TiO_2_-terminated SrTiO_3_(100) [52]. The low Redhead desorption temperature of H_2_O (CO_2_) of 233–283 K (428–523 K) calculated by Baniecki et al. [52] suggests that our surface, which is predominantly TiO_2_-terminated, is cleaned of those adsorbates after annealing, hence the 0.35 eV difference in the WF.

The lower response of TiO to annealing is a hint of the higher adsorption energy of the interacting gases. It suggests the chemisorption of H_2_O and CO_2_ and dissociation of their products, stemming from the high catalytic activity of the TiO phase.

To wrap up our experimental findings of the KPFM study of TiO and SrTiO_3_(100) crystals, a comparison between the obtained work functions as well as the subject literature values is presented in Table 1. The first direct work function measurement of the cubic γ-phase of TiO yields 3.31(21) eV for the as-grown (reduced in UHV at 1150 °C) and 3.75(11) eV for vented and outgassed material, which is most likely close to the value for the pristine TiO crystal. To date, the only values for the TiO WF come from polycrystalline samples (films, nanoparticles) and from the laterally averaging method of UPS, thus this cannot be reasonably compared with our findings. The WF value obtained for the reduced SrTiO_3_(100) surface of 3.12(18) eV follows the trend of a decreasing work function upon reduction and an increasing one due to reoxidation [30]. It is worth mentioning that substantial discrepancies between the measured WF for transition metal oxides, besides the different quality of the samples, could stem from the suboptimal conditions during preparation, e.g., holder materials can act as oxygen getters, influencing the reduction state of a sample [12].

**Table 1:** Comparison between the literature values and the obtained experimental results of TiO and SrTiO_3_ work functions (WF).

Material	Sample	WF [eV]	Method	Ref.

TiO	TiO nanoparticles (14 nm)	3.01	UPS	[44]
	TiO thin polycrystalline film	≈4.5	UPS	[53]
	TiO cubic γ-phase (nanowires):			
	· reduced (UHV, 1150 °C) · air-exposed · air-exposed and annealed	3.31(21) 3.92(17) 3.75(11)	KPFM	this study

SrTiO_3_	SrTiO_3_(100) SrTiO_3_(110) SrTiO_3_(111)	4.13 4.32 4.34	XPEEM	[32]
	SrTiO_3_(100)	4.2	UPS	[33]
	SrTiO_3_(100) reduced at 900 °C	3.478(64)	KPFM	[30]
	TiO_2_-terminated SrTiO_3_(100) SrO-terminated SrTiO_3_(100)	4.2–4.3 2.5–3.0	macroscopic Kelvin probe	[54]
	SrTiO_3_(100) (single crystal):			
	· reduced (UHV, 1150 °C) · air-exposed · air-exposed and annealed	3.12(18) 3.835(72) 3.41(11)	KPFM	this study
	SrTiO_3_(100) BHF-etched, air-annealed (1000 °C) · ϕ_TiO2_ − ϕ_SrO_ difference	≈10 meV	KPFM	[41]
	SrTiO_3_(100) BHF etched, air annealed (1300 °C) · ϕ_TiO2_ − ϕ_SrO_ difference	≈0.5 eV	LEEM	[55]

## Conclusion

We have presented a thorough study of the Kelvin probe force microscopy investigations on the electronic properties of cubic TiO formed on a SrTiO_3_(100) single crystal surface. To our best knowledge, this is the first measurement of the crystalline TiO work function and its dependence on the gaseous pressure of air in comparison to the SrTiO_3_(100) response. The rock-salt cubic γ-phase, with a high crystallographic order, has a work function of 3.31(21) eV with significant variations on the surface, which are related to the different facets exposed. The higher work function of TiO compared to STO(100) is accompanied with a higher conductivity, with an ohmic behavior, which was checked by simultaneous LC-AFM measurements. Full ambient venting results in a 0.6 eV increase in the TiO work function and is a hint of the ongoing catalyzed dissociation of CO_2_ and H_2_O present in ambient air. The work function of TiO decreases by 0.2 eV as a result of 230 °C UHV annealing, being a measure of the amount of weakly adsorbed species. Air exposure of the (√5×√5)R26.6° TiO_2_-terminated SrTiO_3_(100) surface results in a 0.9 eV increase in the work function, which is similar to the increase observed for reoxidation in pure oxygen. Annealing induces a decrease of 0.4 eV as a result of the desorption of weakly bonded species.

Our study also contains an estimation of the FM-KPFM lateral resolution, which enabled reliable measurements of TiO nanowires separated by 40 nm, proving the suitability of the KPFM technique for the investigation of transition metal oxide structures, even with remarkable topographical variations.

In conclusion, the presented results acquired for crystalline TiO and SrTiO_3_ phases provide important information on the work function values themselves but also on their interaction with air and operation upon elevated temperatures.

## Experimental

We investigated Verneuil-grown epitaxy-ready-polished SrTiO_3_(100) crystals, provided by the Crystec company. The crystals were mounted onto Omicron direct heating holders and introduced into the UHV chamber, with a base pressure of 5 × 10^−10^ mbar, maintained by the use of turbo-molecular, ion and sublimation pumps. In order to produce a TiO nanowire network on the surface of STO(100), the extremely low oxygen partial pressure (ELOP) method of perovskite decomposition was adapted (for more information see [12,26]). The SrTiO_3_(100) single crystal (10 × 3 × 0.5 mm^3^) sample was mounted on an n-doped Si(111) single crystal, which acted as an oxygen getter, allowing extremely low effective oxygen partial pressures to be reached on the STO surface (below 10^−20^ mbar). As a result of the high-temperature reduction (1150 °C, 1 h) under such conditions, a macroscopic network of nanowires was formed. After slow cooling down to room temperature, the samples were transferred in situ to the microscope chamber of an Omicron RT STM/AFM system, where KPFM, LC-AFM and STM measurements were performed. KPFM, operating in FM mode, was used with a single-pass method, with three feedback loops maintaining the oscillation amplitude, phase and frequency shift [56]. The real oscillation amplitude was in the range of 10 nm. In order to obtain the CPD signal, an AC-modulated bias was applied to the bottom of the electrode, where a sample was mounted. The modulation frequency and amplitude were set to 315 Hz and 500 mV, respectively.

In order to ensure reproducible FM-KPFM results, two different types of AFM cantilevers were used: PPP-ContPt (PtIr-coated) and PtSi-FM (platinum silicide tips), both from Nanosensors. Such cantilevers are widely used as conducting tips in a contact mode AFM, allowing for a high lateral resolution in conductivity measurements. The remarkable mechanical stability of the selected cantilevers allowed for the noncontact mode measurements (with a Kelvin loop) using the very same tip, maintaining oscillations at the higher harmonics of the fundamental frequency (≈75 kHz). Hence, in order to record current and CPD maps from the very same sample area, KPFM measurements were first performed with the soft cantilever forced to oscillate at higher harmonics, then the tip was retracted tens of nanometers from the surface, all feedback loops were turned down and a contact mode AFM scan was performed when approached with a single loop maintaining a deflection set point of 10–30 mV. The high conductivity of both TiO and STO materials enabled a low sample bias of +1 mV for the LC-AFM measurements to be used.

Real work function values were calculated from recorded CPD maps after calibration against a material of known work function, here highly oriented pyrolytic graphite (HOPG), which was annealed in situ up to 300 °C in order to remove water and other adsorbates. After each set of KPFM measurements, the HOPG surface was scanned employing the same experimental parameters and a *V*_CPD_(HOPG) was obtained. Assuming a HOPG work function of ϕ_HOPG_ = 4.5 eV (subject literature values: 4.6 ± 0.1 eV [57], 4.4 ± 0.1 eV [58]), a work function of TiO/STO was evaluated by applying the equation: ϕ_TiO/STO_ = *V*_CPD_(TiO/STO) – *V*_CPD_(HOPG) + ϕ_HOPG_.

Ambient air exposure was realized by venting the sample in the load-lock and then introducing it again into the UHV system. Subsequent annealing up to 230 °C for 1 h hour enabled the estimation of the content of the weakly adsorbed species at the surface.
